# Quantification of lung surface area using computed tomography

**DOI:** 10.1186/1465-9921-11-153

**Published:** 2010-10-31

**Authors:** Ren Yuan, Taishi Nagao, Peter D Paré, James C Hogg, Don D Sin, Mark W Elliott, Leanna Loy, Li Xing, Steven E Kalloger, John C English, John R Mayo, Harvey O Coxson

**Affiliations:** 1 University of British Columbia James Hogg Research Centre and the Heart and Lung Institute, St. Paul's Hospital; Burrard Street, Vancouver, Canada; 2 UBC Department of Radiology, Vancouver General Hospital; West 12th Ave. Vancouver, Canada; 3 UBC Department of Medicine St. Paul's Hospital; Burrard Street, Vancouver, Canada; 4 UBC Department of Pathology, St. Paul's Hospital; Burrard Street, Vancouver, Canada; 5 UBC Department of Pathology, Vancouver General Hospital, West 12th Ave. Vancouver, Canada

## Abstract

**Objective:**

To refine the CT prediction of emphysema by comparing histology and CT for specific regions of lung. To incorporate both regional lung density measured by CT and cluster analysis of low attenuation areas for comparison with histological measurement of surface area per unit lung volume.

**Methods:**

The histological surface area per unit lung volume was estimated for 140 samples taken from resected lung specimens of fourteen subjects. The region of the lung sampled for histology was located on the pre-operative CT scan; the regional CT median lung density and emphysematous lesion size were calculated using the X-ray attenuation values and a low attenuation cluster analysis. Linear mixed models were used to examine the relationships between histological surface area per unit lung volume and CT measures.

**Results:**

The median CT lung density, low attenuation cluster analysis, and the combination of both were important predictors of surface area per unit lung volume measured by histology (p < 0.0001). Akaike's information criterion showed the model incorporating both parameters provided the most accurate prediction of emphysema.

**Conclusion:**

Combining CT measures of lung density and emphysematous lesion size provides a more accurate estimate of lung surface area per unit lung volume than either measure alone.

## Background

The major pathological components responsible for the decrease in maximal expiratory flow that characterize Chronic Obstructive Pulmonary Disease (COPD) include an increase in airway resistance due to small airway remodeling and obliteration, and a decrease in elastic recoil secondary to the parenchymal tissue destruction which characterizes emphysema [1-3]. Separating the contribution of each of these two components can provide better understanding of the natural history of disease, allow monitoring of disease progression, evaluate the impact of a therapeutic intervention and potentially guide the most appropriate therapeutic target in individual patients. The fact that pulmonary function tests cannot separate these structural changes [4], and because pathological estimates can only do so in surgical or postmortem specimens, has led to attempts to use chest CT scans to measure these changes *in vivo*.

A number of quantitative CT lung densitometry measurements have been employed to measure the extent of emphysema including, 1) the relative area of lung with attenuation values lower than various thresholds [5-10], 2) a specific percentile point on the frequency-attenuation distribution curve [8,9,11], and 3) median lung inflation [12]. However, measurement of lung density may not be the most efficient way to detect emphysema if tissue destruction is accompanied by "remodeling" of the lung parenchyma, such as fibrosis [13-15]. Mishima was the first to introduce cluster analysis of low attenuation areas - a method to measure the size distribution of low attenuation regions [16]. Although validation of this parameter against pathologic standards is controversial [8], we postulated that cluster analysis would supplement lung densitometry in the detection and quantification of emphysema since it is less likely to be affected by tissue deposition.

In the present study, we tested the relationship between the histopathologic reference standard for emphysema - airspace surface area per unit lung volume (SA/V), and two CT measurements: CT lung densitometry (median lung density) and CT cluster analysis. We hypothesized that the combination of the two CT measurements will be superior to the sole use of either in the prediction of SA/V.

## Methods

### Subject Selection

Fourteen subjects (9 men, 5 women) were included in the present study (Table 1). Ten patients underwent lobectomy and four underwent pneumonectomy for lung cancers. Preoperatively, all subjects had spirometry measurements and the diffusing capacity (D*L*co) was measured by the single-breath method of Miller and associates [17]. The study was approved by the hospital and university ethical review boards and all subjects provided written informed consent for the use of all materials and data.

**Table 1:** Subjects Demographics

	Mean ± SD	Range
Age (yrs)	67.0 ± 3.1	61.8 - 72.0
Gender	5 female:9 male	
Smoking (pack yrs)	59.6 ± 44.4	24.8 - 173.0
Height (cm)	169.1 ± 7.2	157.0 - 180.0
Weight (kg)	66.6 ± 12.5	44.0 - 90.0
Post-FEV1%pred (%)	78.7 ± 16.1	46.7 - 114.5
Post-FEV1/FVC	67.5 ± 8.8	45.9 - 79.0
DLCO % pred	70.4 ± 10.3	47.8 - 90.6

Post-FEV1%pred: post-bronchodilator forced expiratory flow in one second/predicted value

Post-FEV1/FVC: post-bronchodilator forced expiratory flow in one second/post-bronchodilator forced vital capacity

D*L*co: Diffusing capacity

### CT Technique

All subjects received a pre-operative, non-contrast helical CT scan in the supine position at the end of full inspiration. 11 subjects were scanned using a GE LightSpeed Ultra CT scanner (General Electric Medical Systems, Milwaukee, WI) with the following settings: 120 kVp, 114 mAs, and 5 mm slices thickness; and 3 subjects were scanned using a Siemens Sensation 16 CT scanner (Siemens Medical Solutions; Erlangen, Germany) with the following parameters: 120 kVp, 115 mAs, and 5 mm slice thickness. The scanners were calibrated regularly using standard water and air phantoms to allow for comparisons between individuals and between scanners.

### Quantitative Histology

Following surgery, the resected specimen was transferred directly from the operating room to the laboratory. The specimen was inflated with Bouin fixative at a constant distending pressure of 25 cm of water and immersed in formalin overnight. After fixation, each specimen was cut into ten slices with 5-8 mm thickness in the axial plane and photographed using a digital camera (Nikon Coolpix, Nikon Corp., Japan). A grid of 2 × 2 cm squares was superimposed over each lung slice, one square was randomly selected and the tissue beneath it was excised, embedded in paraffin, sectioned and stained with haematoxylin and eosin, which resulted in 140 tissue samples in total. Ten random images per histology section were captured using a light microscope (Nikon Microphot) equipped with a digital camera (JVC3-CCD KY F-70, Diagnostic Instruments). The digital images were analyzed using stereologic techniques and a custom program written for Image Pro Plus^® ^digital-image-analysis software (Media Cybernetics) as described elsewhere [18]. Briefly, each image was binarized and a grid of lines was superimposed on the image. The program automatically counts the number of intersections between the superimposed lines and the alveolar walls (i.e., tissue-air interface), the number of line endpoints in one image (i.e., ΣP total), as well as the number of line endpoints that fall on tissue (i.e., ΣPtissue). Surface area per unit lung volume (SA/V) was calculated using the following equations as previously described [12]:

(1) (SA/V)=surface density of the tissue–air interface×volume fraction of tissue,

in which, surface density of the tissue-air interface [19]:

(2) Sv(tis)=(4/L)×(ΣI/ΣPtissue)=2/mean linear intercept

where L = the length of the grid unit line, ΣI = the number of intersections counted, ΣPtissue is the number of line end points that fall on tissue.

The volume fraction of tissue:

(3) Vv(tis)=ΣP tissue/ΣP total,

where ΣP total is the number of line end points counted in one image.

SA/V for each of the samples was corrected for shrinkage. The shrinkage factor was determined by measuring the length of one side of the blocks prior to fixation processing and then dividing by the length of that side of the cut sections post-fixation (shrinkage factor: 1.30 ± 0.13)

### Quantitative CT

**Figure 1:**
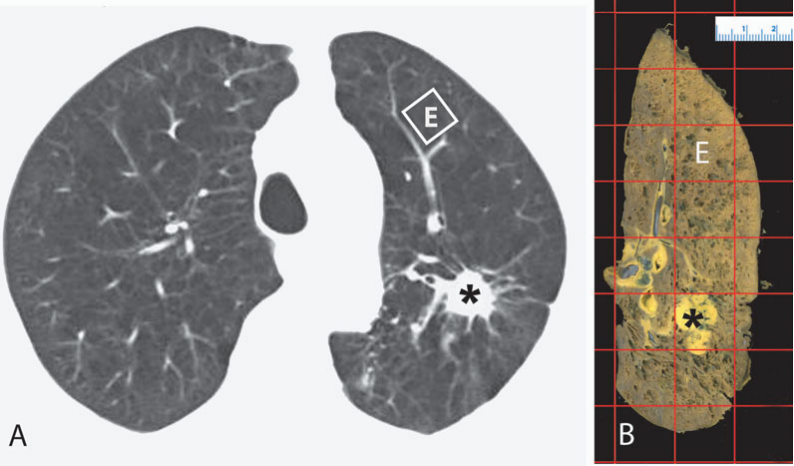
**Matching CT Images and Lung Specimens**. A CT image of a representative subject is shown in Figure 1A and the corresponding slice of the resected specimen is shown in Figure 1B. For reference and orientation, the tumor is marked by a star (_*_). A grid is superimposed over the fixed lung slice (Figure 1B) and a 2 × 2 cm square section (square E) is randomly selected for histological processing and measurement of surface area per unit lung volume (SA/V). The corresponding region (square E) on CT is then identified (Figure 1A); the CT median lung density and the CT cluster analysis value D are obtained in the region of interest using the computer program (*EmphylxJ*). The size of the square E on CT has been corrected for lung inflation to match the size of the histological specimen.

The region of lung where the histology samples were taken was identified on the CT image by comparing anatomic landmarks on the cut surface of the gross lung specimen and CT images as shown in Figure 1. The difference in lung inflation between the *in vivo *and *in vitro *state was determined by comparing the area of the cut surface on the lung specimen, measured using *ImageJ*, (Rasband, W.S., ImageJ, U. S. National Institutes of Health, Bethesda, Maryland, USA, http://rsb.info.nih.gov/ij/, 1997-2007) to the area of the lung on the *in vivo *CT image measured using custom software (EmphylxJ, UBC James Hogg Research Centre, Vancouver, B.C, http://www.flintbox.com) as described elsewhere [20]. Then, a square, size-corrected for inflation was superimposed upon the CT image. For each voxel within that square, the apparent X-ray attenuation value (Hounsfield Unit, HU) was obtained and converted to gravimetric density (g/ml) by adding 1000 to the HU value and dividing by 1000 [21]. The median CT lung density value was chosen from the frequency distribution curve of lung density within each square since the curve is skewed to the right [12]. We estimated the distribution of sizes of the emphysematous lesions within each square using a low attenuation cluster analysis [16,22]. In the low attenuation cluster analysis the inverse slope of the log-log relationship of the size of the low attenuation cluster (number of contiguous voxels <-856 HU) versus the number of clusters of that size is the power-law exponent (D). -856HU was chosen to identify "emphysematous" because it converts to 6.0 ml/g, which has been previously shown to represent the boundary between normal and mildly emphysematous lung [12] (See additional file 1: Converting 6.0 ml/g to -856HU).

### Statistical Analysis

The primary outcome was the histologically measured SA/V and the independent variables included the median CT lung density and the CT cluster analysis value D. We used a linear mixed model (the REstricted Maximum Likelihood method, REML) to incorporate the within subject variance of the measurements since ten measurements were made from each lung specimen [23], and we examined the association between the outcome and the two independent variables with the gender, age and patient's body mass index (BMI) being covariates. To test whether CT cluster analysis could supplement lung densitometry (i.e., median lung density) in detecting histological emphysema, we compared the prediction of SA/V using median CT lung density or the CT cluster analysis value D to a third model, which incorporated both variables using Akaike's Information Criterion (AIC) based on the Maximum Likelihood Estimation [24]. The model with the smallest AIC value is considered to be the best model [25]. Analyses were performed using SAS version 9.1 (Carey, N.C.). Statistical significance was defined at a p-value less than 0.05. Continuous variables are expressed as mean ± SD.

## Results

### Subject Characteristics

The subject demographics are shown in Table 1. The level of airway obstruction of the subjects was relatively mild with only one subject in stage 3 according to the Global Initiative for Obstructive Lung Disease (GOLD) categories [26]. Five subjects were stage 2, two stage 1, and the remaining six subjects had normal lung function.

### Quantitative Histology and Quantitative CT Measurements

The histological measurements of SA/V and quantitative CT measurements for all 140 tissue samples from 14 cases are summarized in Table 2. These data show that there is a wide variation in both histological and quantitative CT measurements within each individual.

**Table 2:** Histological and Quantitative CT Measurements for 140 Tissue Samples from 14 Subjects

Subject	Histology-SA/V **(cm**^**2**^**/cm**^**3**^**)**	Median CT lung density (g/ml)	Low Attenuation Cluster Analysis (D)
1	161.4 ~ 275.3	5.6 ~ 7.9	0.2 ~ 1.1
2	175.1 ~ 265.6	6.5 ~ 7.5	0.1 ~ 0.7
3	102.5 ~ 215.3	5.9 ~ 8.3	0.2 ~ 0.9
4	182.7 ~ 438.6	4.2 ~ 5.8	0.6 ~ 2.5
5	39.2 ~ 122.2	11.7 ~ 39.1	0.1 ~ 0.3
6	172.0 ~ 253.9	4.7 ~ 6.9	0.2 ~ 1.2
7	84.3 ~ 171.3	8.2 ~ 14.8	0.1 ~ 0.4
8	171.9 ~ 289.2	5.6 ~ 9.3	0.3 ~ 1.2
9	90.6 ~ 260.1	7.3 ~ 13.8	0.1 ~ 0.6
10	227.4 ~ 464.1	2.9 ~ 4.8	1.1 ~ 2.0
11	141.7 ~ 256.5	3.2 ~ 6.7	0.6 ~ 2.0
12	320.2 ~ 445.6	3.6 ~ 5.9	0.9 ~ 2.2
13	78.0 ~ 248.3	6.1 ~ 14.8	0.1 ~ 0.7
14	237.6 ~ 332.6	4.8 ~ 6.3	0.6 ~ 2.0

**Figure 2:**
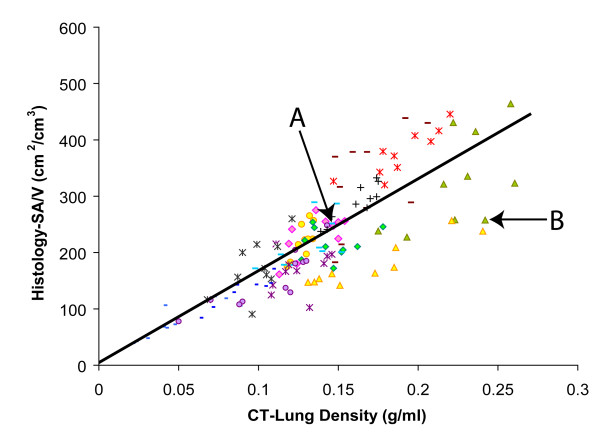
**Association between the Histological SA/V and CT Median Lung Density**. There is a significant association between the SA/V (cm^2^/cm^3^) measured histologically and the CT median lung density (g/ml) (r = 0.82, p < 0.0001). All subjects are shown using different symbols. Data point A and B refer to samples with comparable SA/V value but very different CT density measurement (sample A: SA/V = 247 cm^2^/cm^3^, CT density = 0.14 g/ml; sample B: SA/V = 258 cm^2^/cm^3^, CT density = 0.24 g/ml). A and B refer to the same samples in Figure 2, 3, and 5.

**Figure 3:**
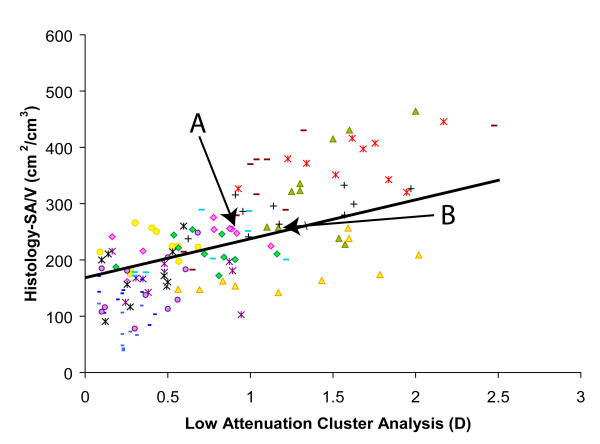
**Association between the Histological SA/V and CT Cluster Analysis Value D**. There is a significant association between the SA/V (cm^2^/cm^3^) measured histologically and the CT cluster analysis D value (r = 0.74, p < 0.0001). All subjects are shown using different symbols. Data point A and B have comparable value for SA/V and CT cluster analysis (sample A: SA/V = 247 cm^2^/cm^3^, D = 0.91; sample B: SA/V = 258 cm^2^/cm^3^, D = 1.17). A and B refer to the same samples in Figure 2, 3, and 5.

Linear mixed models showed that the median CT lung density and the CT cluster analysis value D were significantly associated with histological SA/V (both p < 0.0001) (Figures 2 and 3). The prediction equations of SA/V using CT lung density alone, CT cluster analysis alone, and the combination of these two measurements were:

SA/V = 4.62 + 1631.99 × median CT lung density;

SA/V = 168.44 + 69.21 × CT cluster analysis value D;

SA/V = 6.04 + 1597.05 × median CT lung density + 11.19 × CT cluster analysis value D.

A comparison of the three models using the Akaike's Information Criterion showed that the model incorporating both CT lung density and low attenuation cluster analysis yielded the smallest AIC value indicating that it is the best model for predicting SA/V (the AIC was 904 for CT lung density alone, 927 for CT cluster analysis alone and 897 for the model incorporating both variables).

## Discussion

**Figure 4:**
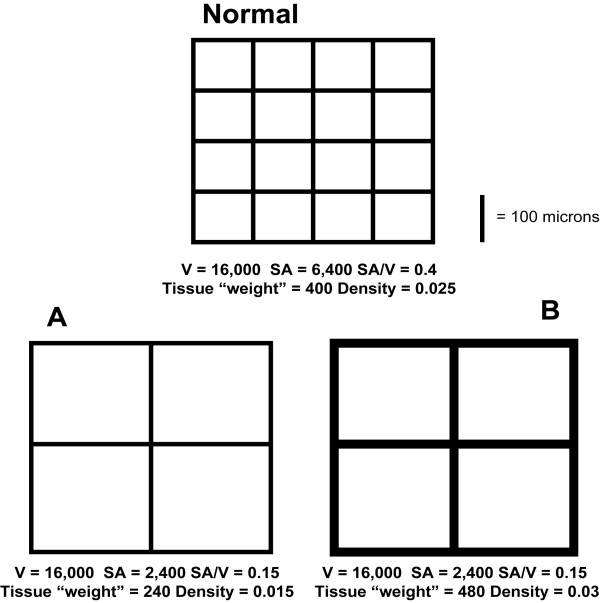
**A Schematic Showing the Relationship between Lung SA/V and Density under two scenarios**. The top panel represents normal lung architecture with the dimensions of each "alveolus" being 100 × 100 μm yielding a total volume of the "lung" = 16,000 μm^3 ^with a surface area of 6,400 μm^2 ^and a SA/V of 0.4. If we assign a mass of 10 units to each 100 μm length of "alveolar wall" this "lung" has a mass of 400 units and a density of 0.025 units/μm^3 ^(= 400 units/16,000 μm^3^). In A, the volume and thickness of the "alveolar walls" remains the same as those in "normal lung architecture" but the surface area is decreased due to destruction of "alveolar walls". In this scenario, the reduction in SA/V and density are proportional. However in scenario B, the thickness of the "alveolar walls" is doubled therefore increasing the mass. The resultant SA/V is the same as in A whereas the density is higher than in A and even higher than the Normal. Thus if there is addition of tissue, the relationship between SA/V and density is disrupted.

The most important finding of the present study is that although CT lung densitometry (i.e., median lung density in the current study) was a valid estimate of the histological measurement of airspace enlargement and/or alveolar wall destruction (airspace surface area per unit lung volume, SA/V), its accuracy was significantly improved by combining it with CT cluster analysis of lower attenuation areas. Basing an estimate of emphysema solely on a measure of lung density assumes that the decrease in alveolar surface area which accompanies emphysema is mirrored by a proportional reduction in lung tissue mass. Although it is clear that tissue destruction is part of the process, there is increasing evidence that emphysema is also accompanied by "remodeling" of the lung parenchyma which may be associated with fibrosis [13-15]. The extent of this "remodeling" will confound the relationship between lung density and SA/V. This phenomenon is illustrated in Figure 4. In this schematic, normal lung architecture (Normal) and two examples of "emphysema" (A and B) are shown. In example A, there is a loss of alveolar walls with a corresponding loss of lung mass. In example B, there is a similar loss of the number of alveolar walls but a thickening of the retained alveolar walls such that the mass of the lung is comparable to Normal and greater than in A although both A and B have comparable loss in lung SA/V.

**Figure 5:**
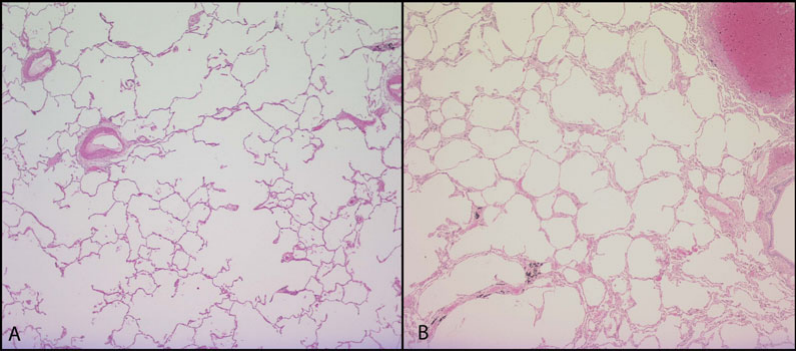
**Hematoxylin and Eosin-stained Images of Tissue Samples A and B in Figures 2, 3**. The tissue shown in A has a SA/V of 247 mm^2^/mm^3 ^and a CT density of 0.14 g/ml while the area in B has a SA/V of 258 mm^2^/mm^3 ^and a CT density of 0.24 g/ml. Thus despite comparable SA/V, there is a substantial difference in CT density due to the deposition of extracellular matrix in B. On the other hand, CT cluster analysis (i.e., value D), which relies solely on the size of the low attenuation areas, was comparable in these two regions (0.97 in A and 1.17 in B).

CT cluster analysis of low attenuation areas is a method to describe and quantify the distribution of emphysematous spaces by determining whether low attenuation voxels are clustered into large lesions or present as diffuse small ones. It has been shown that there is an inverse power law relationship between the size and number of clusters where the slope of this relationship (D) becomes smaller with increasing lesion size [16]. This variable is less likely to be affected by the accumulation of connective tissue that may accompany emphysema since it measures clustering of low attenuation areas. Examples of these theoretical considerations were observed in our data. For example, points A and B in Figure 2 and 3 represent two samples with comparable values for histological SA/V and CT cluster analysis but very different CT lung density. The examination of the histology in these two samples shown in Figure 5 is consistent with the theory illustrated in Figure 4. For sample B CT cluster analysis provides a more accurate estimate of histological SA/V than does CT lung density, because tissue deposition accompanies tissue destruction. Additionally the cluster analysis likely detects true tissue destruction with the formation of low attenuation areas larger than single CT voxels while measures of density can be affected by simple hyperinflation of lung tissue without alveolar wall destruction. Such hyperinflation may be a precursor of the tissue destruction which characterizes emphysema but would have less effect on the histological surface area to volume ratio than true tissue disruption.

The current data also suggest that the cluster analysis value D, per se, is a valid quantitative CT estimate of emphysema because it significantly, and independently, correlated with the histological measurement of surface area per unit lung volume (Figure 3). This finding is at variance with that of Madani et al [8]. We think this discrepancy might be because we chose a different HU cutoff to define the "low attenuation cluster". Madani et al chose -960HU and 1^st ^percentile point as the cutoff whereas we used a relatively higher HU value: -856HU. As we explained in the methods section that -856 HU is converted from a lung tissue inflation value of 6.0 ml/g, which was previously shown to represent the boundary between normal and mild emphysematous lung [12].

In the current study, we chose surface area per unit lung volume (i.e., SA/V) as the histological reference. This variable has been shown to separate normal lung from emphysematous tissue [12], and its calculation (Equation 1 and 2) is linearly related to the mean linear intercept (i.e., *Lm*), which has been used by other groups to estimate emphysema microscopically [9].

**Figure 6:**
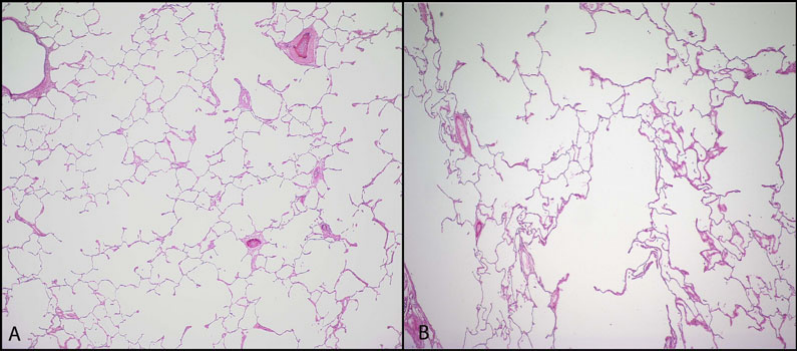
**Heterogeneity of Lung Tissue Destruction**. Examples of hematoxylin and eosin-stained images of tissue samples taken from the same individual but different lung regions. A: Normal tissue with SA/V = 439 cm^2^/cm^3^, tissue density = 0.19 g/ml, B: emphysematous tissue with SA/V = 183 cm^2^/cm^3^, tissue density = 0.14 g/ml.

One challenge for validation of CT measurements is the marked heterogeneity of the emphysematous process. Even in lungs severely affected by emphysema, some regions still maintain normal architecture making sampling for pathological examination critical as shown in Figure 6. In many of the previous validation studies, including our previous work, the commonly applied approach is to randomly sample tissue from lungs, calculate the averaged value from these random samples to obtain one single histological measurement for each subject, and compare this value to one single CT measurement obtained from the whole lung of that subject [6,8,9,11,12]. However, by doing so, the CT measurement is global, incorporating all regions, diseased or relatively normal, whereas the histological measurement is averaged from a limited number of samples taken from different regions of the surgically resected lungs. In the present study, we have refined this approach by using a modified computer program, which enables us to obtain regional CT measurements from the exact regions of the lung where the histological measurements were taken and compare this regional CT measurement to the histological measurement of the same region. We think this precise matching can provide a more accurate comparison between CT and histological measurements. Also, in this way, we were testing our hypothesis in 140 tissue samples rather than in 14 subjects. Nevertheless, we cannot consider 140 tissue samples as 140 independent samples since ten samples were taken from each individual. Therefore, in the statistical analysis, we applied a linear mixed modeling approach to account for the random effects arising from inter-individual variance and to obtain prediction equations at the group level [23].

This study has some limitations. First, in the current study, we only used one CT densitometry measurement, median lung density. While Gevenois has shown using thin slice CT scans (1 mm) that -950 HU detects both macroscopic and microscopic emphysema they also showed that using this cut-off 6.8% would be the upper limit of normal and therefore the threshold between normal and diseased [6]. However, previous studies using thick slice CT scans shows that threshold cut-offs such as -910 HU only pick up large emphysematous holes in the lung [27] while a threshold of -856 HU estimates the small holes [12]. Therefore, with this data in mind, we chose the mean lung density threshold, because of the small size of pathologic specimens (2 × 2 cm^2^) that we were comparing to the thick slice CT values and the relatively mild degree of emphysema present in our subjects and specimens. We cannot comment on the supplementary role of CT cluster analysis to other more traditional whole lung CT densitometry measurements of emphysema, such as low attenuation area and percentile point, etc. However, we believe it is reasonable to assume that CT cluster analysis would supplement the other CT densitometry measurements since all such measurements rely on choosing a cutoff value from the X-ray attenuation distribution histogram, either along the X axis (i.e., low attenuation area) or along the Y axis (i.e., percentile point). The extent, to which, CT cluster analysis supplements the different CT densitometry measurements might vary depending on the threshold use and, therefore, further studies including other densitometry measurements may provide more information. Secondly, we used -856HU, based on our previous experience with thick slice CT scans that identified this HU threshold as effective in identifying mild emphysematous areas [12]. We realize that CT scan slices in our previous study were of 10 mm thickness whereas in the current study were of 5 mm slice thickness. Due to limitations in CT scanner technology, we are not able to test whether this threshold is equally effective using either slice thickness. Lastly, the pre-surgery CT images were acquired using two different CT scanners could have introduced errors in CT lung density measurement. However since the X-ray radiation dose is similar (120 kVp and 114 mAs on GE scanner; 120 kVp and 115 mAs on Siemens scanner), we believe this effect is small. Moreover we have previously shown that CT densitometry measurements using similar acquisition protocols are comparable between these CT scanners [20].

The difference in Akaike's Information Criterion (AIC) between the models appears small but this does not mean that the added information of the combined model is small. The AIC cannot be interpreted using a traditional "hypothesis testing" statistical paradigm. It does not generate a P value, does not reach conclusions about "statistical significance", and does not "reject" any model. AIC determines how well the data supports each model, taking into account both the goodness-of-fit (sum-of-squares) and the number of parameters in the model. Ultimately, the model with the smallest AIC is considered the best, although the AIC value itself is not meaningful [28].

In conclusion, the results of this study show that an accurate comparison between CT and histological measurements can be achieved by precisely mapping the location of the histological sample to its *in vivo *location on the CT. In addition, the CT cluster analysis value D can supplement CT densitometry in detecting and quantifying emphysema. The additional benefit may be due to the fact that cluster analysis is more sensitive to true tissue destruction and immune to the artifact caused by the deposition of connective tissue that may accompany the emphysematous process.

## Competing interests

PD Paré is the principal investigator of a project funded by GSK to develop CT based algorithms to quantify emphysema and airway disease in COPD. With collaborators he has received ~ $300,000 to develop and validate these techniques. These funds he have been applied solely to the research to support programmers and technicians. Peter Pare was also PI of a Merck Frosst supported research program to investigate gene expression in the lungs of patients who have COPD. He and collaborators have received ~$200,000 for this project. These funds have supported the technical personnel and expendables involved in the project. PP has established a new contract with Merck to discover genetic predictors of gene expression in lung tissue. With collaborators he will receive $95,000 over the next year to do this work. The funds will support personnel and buy supplies. PP sits on an advisory board for Talecris Biotherapeutics who make anti-one antitrypsin replacement therapy.

JC Hogg has served as a consultant, given lectures and participated in advisory boards of several major pharmaceutical companies in the past five years. The total reimbursement for these activities is less than $20000.00. His University (UBC) has also received industry sponsored grants from GSK and Merck on which he serve as the PI.

DD Sin has received research funding from GlaxoSmithKline and AstraZeneca for projects on chronic obstruction pulmonary disease. DD Sin has also received honoraria for speaking engagements for talks on COPD sponsored by these organizations.

HO Coxson received $4800 in 2006 - 2008 for serving on the steering committee for the ECLIPSE project for GSK. In addition HC is the co-investigator on two multi-center studies sponsored by GSK and has received travel expenses to attend meetings related to the project. HC has three contract service agreements with GSK to quantify the CT scans in subjects with COPD and a service agreement with Spiration Inc to measure changes in lung volume in subjects with severe emphysema. A percentage of HC's salary between 2003 and 2006 (15,000 US $/year) derives from contract funds provided to a colleague PD Pare by GSK for the development of validated methods to measure emphysema and airway disease using computed tomography. HC is the co-investigator (DD Sin PI) on a Canadian Institutes of Health - Industry (Wyeth) partnership grant.

R Yuan, T Nagao, WM Elliott, L Loy, L Xing, S Kalloger, J English, and J Mayo have no competing interests in the content of this manuscript.

## Authors' contributions

RY and TN carried out the quantitative CT analysis. WME and LL carried out the quantitative histological analysis. DS and LX performed the statistical analysis. PP is the principal investigator of the project, obtained funding for and supervised the project. PP, JH, and HC participated in the design of the study. RY, PP, JH and HC drafted the manuscript. SK, JE and JM participated in the coordination of the study and helped to draft the manuscript. All authors read and approved the final manuscript.

## Supplementary Material

Additional file 1 **Conversion of 6.0 ml/g to -856HU**. This file outlines the method to convert lung inflation values, measured as ml of gas per g tissue, into X-ray attenuation values. Click here for file

## References

[B1] Burrows B Knudson RJ Cline MG Lebowitz MD Quantitative relationships between cigarette smoking and ventilatory function Am Rev Respir Dis 1977 115 195 205 842934 10.1164/arrd.1977.115.2.195

[B2] Hogg JC Macklem PT Thurlbeck WM Site and nature of airway obstruction in chronic obstructive lung disease N Engl J Med 1968 278 1355 1360 10.1056/NEJM196806202782501 5650164

[B3] Macklem PT Mead J Resistance of central and peripheral airways measured by a retrograde catheter J Appl Physiol 1967 22 395 401 4960137 10.1152/jappl.1967.22.3.395

[B4] Fraser RS Paré PD Colman NC Muller NL Diagnosis of Diseases of the Chest 1999 Fourth Philadelphia: Saunders

[B5] Bankier AA De Maertelaer V Keyzer C Gevenois PA Pulmonary emphysema: subjective visual grading versus objective quantification with macroscopic morphometry and thin-section CT densitometry Radiology 1999 211 851 858 10352615 10.1148/radiology.211.3.r99jn05851

[B6] Gevenois PA De Vuyst P de Maertelaer V Zanen J Jacobovitz D Cosio MG Yernault JC Comparison of computed density and microscopic morphometry in pulmonary emphysema Am J Respir Crit Care Med 1996 154 187 192 8680679 10.1164/ajrccm.154.1.8680679

[B7] Hayhurst MD MacNee W Flenley DC Wright D McLean A Lamb D Wightman AJ Best J Diagnosis of pulmonary emphysema by computerised tomography Lancet 1984 2 320 322 10.1016/S0140-6736(84)92689-8 6146866

[B8] Madani A Van Muylem A de Maertelaer V Zanen J Gevenois PA Pulmonary emphysema: size distribution of emphysematous spaces on multidetector CT images-comparison with macroscopic and microscopic morphometry Radiology 2008 248 1036 1041 10.1148/radiol.2483071434 18710992

[B9] Madani A Zanen J de Maertelaer V Gevenois PA Pulmonary emphysema: objective quantification at multi-detector row CT--comparison with macroscopic and microscopic morphometry Radiology 2006 238 1036 1043 10.1148/radiol.2382042196 16424242

[B10] Muller NL Staples CA Miller RR Abboud RT "Density mask". An objective method to quantitate emphysema using computed tomography Chest 1988 94 782 787 10.1378/chest.94.4.782 3168574

[B11] Gould GA MacNee W McLean A Warren PM Redpath A Best JJ Lamb D Flenley DC CT measurements of lung density in life can quantitate distal airspace enlargement - an essential defining feature of human emphysema Am Rev Respir Dis 1988 137 380 392 3341629 10.1164/ajrccm/137.2.380

[B12] Coxson HO Rogers RM Whittall KP D'Yachkova Y Pare PD Sciurba FC Hogg JC A quantification of the lung surface area in emphysema using computed tomography Am J Respir Crit Care Med 1999 159 851 856 10051262 10.1164/ajrccm.159.3.9805067

[B13] Lang MR Fiaux GW Gillooly M Stewart JA Hulmes DJ Lamb D Collagen content of alveolar wall tissue in emphysematous and non-emphysematous lungs Thorax 1994 49 319 326 10.1136/thx.49.4.319 8202900 PMC475363

[B14] Tonelli M Stern EJ Glenny RW HRCT evident fibrosis in isolated pulmonary emphysema J Comput Assist Tomogr 1997 21 322 323 10.1097/00004728-199703000-00031 9071310

[B15] Cardoso WV Sekhon HS Hyde DM Thurlbeck WM Collagen and elastin in human pulmonary emphysema Am Rev Respir Dis 1993 147 975 981 8466136 10.1164/ajrccm/147.4.975

[B16] Mishima M Hirai T Itoh H Nakano Y Sakai H Muro S Nishimura K Oku Y Chin K Ohi M Complexity of terminal airspace geometry assessed by lung computed tomography in normal subjects and patients with chronic obstructive pulmonary disease Proc Natl Acad Sci USA 1999 96 8829 8834 10.1073/pnas.96.16.8829 10430855 PMC17692

[B17] Miller A Thornton JC Warshaw R Anderson H Teirstein AS Selikoff IJ Single breath diffusing capacity in a representative sample of the population of Michigan, a large industrial state. Predicted values, lower limits of normal, and frequencies of abnormality by smoking history Am Rev Respir Dis 1983 127 270 277 6830050 10.1164/arrd.1983.127.3.270

[B18] Hogg JC Chu F Utokaparch S Woods R Elliott WM Buzatu L Cherniack RM Rogers RM Sciurba FC Coxson HO Pare PD The nature of small-airway obstruction in chronic obstructive pulmonary disease N Engl J Med 2004 350 2645 2653 10.1056/NEJMoa032158 15215480

[B19] Howard CV Reed MG Unbiased Stereology: Three-Dimensional Measurement in Microscopy, Second Edition Summary 2004 Second Liverpool, UK: Taylor & Francis Inc

[B20] Yuan R Mayo JR Hogg JC Pare PD McWilliams AM Lam S Coxson HO The Effects of Radiation Dose and CT Manufacturer on Measurements of Lung Densitometry Chest 2007 132 617 623 10.1378/chest.06-2325 17573501

[B21] Hedlund LW Vock P Effmann EL Evaluating lung density by computed tomography Semin Respir Med 1983 5 76 87 10.1055/s-2007-1011435

[B22] Coxson HO Whittall KP Nakano Y Rogers RM Sciurba FC Keenan RJ Hogg JC Selection of patients for lung volume reduction surgery using a power law analysis of the computed tomographic scan Thorax 2003 58 510 514 10.1136/thorax.58.6.510 12775863 PMC1746695

[B23] Feldman HA Families of lines: random effects in linear regression analysis J Appl Physiol 1988 64 1721 1732 3379003 10.1152/jappl.1988.64.4.1721

[B24] Verbeke G Molenberghs G Linear Mixed Models for Longitudinal Data 2000 Springer-Verlag New York

[B25] Ljung L System Identification: Theory for the User 1999 Upper Saddle River, NJ: Prentice-Hal PTR

[B26] Rabe KF Hurd S Anzueto A Barnes PJ Buist SA Calverley P Fukuchi Y Jenkins C Rodriguez-Roisin R van Weel C Zielinski J Global strategy for the diagnosis, management, and prevention of chronic obstructive pulmonary disease: GOLD executive summary Am J Respir Crit Care Med 2007 176 532 555 10.1164/rccm.200703-456SO 17507545

[B27] Miller RR Muller NL Vedal S Morrison NJ Staples CA Limitations of computed tomography in the assessment of emphysema Am Rev Respir Dis 1989 139 980 983 2930075 10.1164/ajrccm/139.4.980

[B28] Lindsey JK Jones B Choosing among generalized linear models applied to medical data Stat Med 1998 17 59 68 10.1002/(SICI)1097-0258(19980115)17:1<59::AID-SIM733>3.0.CO;2-7 9463849

