# Supplementary material for: Quantification of lung surface area using computed tomography
Source: Respir Res. 2010 Oct 31;11(1):153. doi: 10.1186/1465-9921-11-153 (PMC2976969; doi:10.1186/1465-9921-11-153)
Supplement: Additional file 1 — Conversion of 6.0 ml/g to -856HU. This file outlines the method to convert lung inflation values, measured as ml of gas per g tissue, into X-ray attenuation values. [file 1465-9921-11-153-S1.DOC]

**Additional file 1: Converting 6.0ml/g to -856HU**

On CT, the density of the lung (tissue and airspace) was estimated by adding 1,000 to the Hounsfield Units of each voxel, and dividing by 1,000 [1] as shown in Equation 1.

CT Density, g/ml = (HU +1000)/1000 (1)

Lung weight was estimated by multiplying the mean lung density by the volume of the whole lung (tissue and airspace), which was calculated by summing the voxel dimensions in each slice.

The volume of gas per gram of tissue for each voxel was calculated according to Equation 1 [2]:

$\frac{ml(gas)}{g(tissue)}=$ *Specific volume _(tissue & gas)_ - Specific volume _(tissue)_* (2)

where specific volume is the inverse of density. The density of the lung (tissue and gas) was measured from the CT using Equation 1, and the density of tissue was assumed to be 1.065 g/ml [3].

Therefore, Equation 2 can be rewritten as following:

$\frac{ml(gas)}{g(tissue)}=$*1/ CT Density _(tissue & gas)_ -1/ Density _(tissue)_*

$=$ 1000/ (HU + 1000) – 1/1.065 (3)

If volume of gas per gram of tissue is 6.0, the corresponding HU calculated according to Equation 3 is -856.

**References:**

1. Hedlund LW, Vock P, Effmann EL: **Evaluating lung density by computed tomography**. *Semin Respir Med* 1983, **5**:76–87.

2. Coxson HO, Rogers RM, Whittall KP, D'Yachkova Y, Pare PD, Sciurba FC, Hogg JC: **A quantification of the lung surface area in emphysema using computed tomography**. *Am J Respir Crit Care Med* 1999, **159**(3):851-856.

3. Hogg JC, Nepszy S: **Regional lung volume and pleural pressure gradient estimated from lung density in dogs**. *J Appl Physiol* 1969, **27**(2):198-203.
